# Balsam fir (*Abies balsamea*) needles and their essential oil kill overwintering ticks (*Ixodes scapularis*) at cold temperatures

**DOI:** 10.1038/s41598-022-15164-z

**Published:** 2022-07-29

**Authors:** Shelley A. Adamo, Amal El Nabbout, Laura V. Ferguson, Jeffrey S. Zbarsky, Nicoletta Faraone

**Affiliations:** 1 Department Psychology and Neuroscience, Dalhousie University, Halifax, NS B3H3X5 Canada; 2 Department Biology, Acadia University, Wolfville, Canada; 3 Department Chemistry, Acadia University, Wolfville, Canada

**Keywords:** Entomology, Climate-change ecology, Agroecology

## Abstract

The blacklegged tick, *Ixodes scapularis*, vectors *Borrelia burgdorferi*, a bacterium that causes Lyme Disease. Although synthetic pesticides can reduce tick numbers, there are concerns about their potential effects on beneficial insects, such as pollinators. Plant-based pest control agents such as essential oils could provide an alternative because they have low environmental persistency; however, these products struggle to provide effective control. We found a new natural acaricide, balsam fir (*Abies balsamea*) needles, that kill overwintering *I. scapularis* ticks. We extracted the essential oil from the needles, analyzed its chemical composition, and tested it for acaricidal activity. We placed ticks in tubes with substrate and positioned the tubes either in the field or in incubators simulating winter temperatures. We added balsam fir essential oil, or one of the main components of balsam fir essential oil (i.e., ß-pinene), to each tube. We found that both the oil and ß-pinene kill overwintering ticks. Whole balsam fir needles require several weeks to kill overwintering ticks, while the essential oil is lethal within days at low temperatures (≤ 4 °C). Further, low temperatures increased the efficacy of this volatile essential oil. Higher temperatures (i.e., 20 °C) reduce the acaricidal effectiveness of the essential oil by 50% at 0.1% v/v. Low temperatures may promote the effectiveness of other natural control products. Winter is an overlooked season for tick control and should be explored as a possible time for the application of low toxicity products for successful tick management.

## Introduction

The tick *Ixodes scapularis* is a vector of *Borrelia burgdorferi*, a bacterium that causes Lyme Disease^1^. Although synthetic pesticides, such as pyrethroids like permethrin and deltamethrin^2^, are currently used to kill ticks^3^, concerns over the deleterious effects of pesticides on the environment have spurred the development of acaricidal compounds derived from plants, such as essential oils^2,4,5^. Essential oils appear to be less toxic to beneficial insects, such as pollinators^6–8^. Although essential oils can kill *I. scapularis*^2,5^, they have had limited success in the field^4,9–11^. Some of the very attributes that reduce their environmental impact, such as low persistence, also reduce their effectiveness^9,12^. In this paper, we demonstrate that it may be possible to boost the effectiveness of essential oils by applying them during the winter season when temperatures are low. In fact, essential oils have greater persistence at lower temperatures^13^, making them appealing to be used during colder months.

Synthetic acaricides (i.e., tick-killing compounds) are applied during the ticks’ active season—i.e., spring, summer and fall^10^. However, all three stages of *I. scapularis* ticks (e.g., larvae, nymph and adult) can be found during the winter^14^. Low winter temperatures are stressful for terrestrial arthropods^15^, and *I. scapularis* is susceptible to stressors such as chill injury, desiccation, and inoculative freezing; thus, cold and dry winters can be lethal^16,17^. To survive these stresses, *I. scapularis* ticks must use resources to increase the production of protective molecules, such as antifreeze proteins^18^. During this stressful time, ticks may be more susceptible to a second stressor (e.g., a toxic compound). Animals often respond less effectively when facing two stressors simultaneously^19,20^. Therefore, it is plausible that compounds with minimal toxicity to ticks at higher temperatures may become more lethal in the cold.

One important behavioural strategy *I. scapularis* ticks use to survive through the winter is burrowing into leaf litter^21^. Leaf litter provides an insulated and more humid environment than that offered by an exposed site^17^. Indeed, ticks overwintering under leaf litter can withstand air temperatures well below 0°C^21,22^. The insulation provided by leaf litter results in soil temperatures that are warmer than air temperatures during the winter^23^ with rates of mortality similar to those in summer conditions^16^. For example, leaf litter allows ticks (such as nymphs^14^, larvae/nymphs^22^) to have greater than 80% survival over the winter months, although overwintering success can vary widely from year to year^24,25^. Given that winter conditions typically persist for four to five months in Canada and parts of the American Northeast, ticks in these areas spend considerable time in contact with plant material in the leaf litter, and thus the type of leaf litter surrounding ticks could impact their winter mortality^26^. Some have suggested that coniferous forests are likely to provide less insulating leaf litter, leading to reduced overwintering survival^17^. However, the survival of engorged larval *I. scapularis* ticks was the same whether they overwintered in deciduous or coniferous wood stands^26^. Similarly, there was no difference in the overwintering survival of unfed, adult female ticks in a maple forest, white pine stand, or oak savannah environment, although in these studies the bottom substrate of the microcosm was Kentucky bluegrass sod (*Poa pratensis*)^24,25^. Therefore, although leaf litter is important for tick overwintering survival^21^, there is no direct evidence that the type of leaf litter is important. However, leaf litter is also highly variable in depth and composition, across even relatively small areas, suggesting that microenvironments can change rapidly within a short distance^27^. These results suggest that further study of the effect of leaf litter on tick overwintering survival is warranted.

Because of the need for inexpensive, and potentially more environmentally-friendly methods of tick control, we decided to revisit the question of whether the type of leaf litter can reduce overwintering survival in *I. scapularis*. Anecdotal observations by one of us (SAA) suggested that ticks did not successfully overwinter under balsam fir (*Abies balsamea*) needles. We also chose this particular conifer because it is not a common tree in the historical home range of *I. scapularis. Ixodes scapularis* ticks may be more likely to be sensitive to compounds with which they have not co-evolved. Balsam fir is common in Eastern Canada and in the extreme northern part of the eastern half of the US, but it is rare further south^28^ in the region thought to be the ancestral range of *I. scapularis*^29^. We tested the acaricidal activity of balsam fir needles and the essential oil extracted from those needles, against overwintering *I. scapularis* ticks using both lab and field studies. We determined the essential oil composition of our local balsam fir needles and tested whether its main component, β-pinene, plays a role in killing ticks. We also tested whether the acaricidal effect was temperature-dependent.

## Results

### Balsam fir needles kill overwintering ticks under both field and lab conditions

Locally collected adult ticks were placed in individual tubes with vermiculite and sand, topped with 1 cm of balsam fir needles or 1 cm of maple/oak leaf litter (approximately 2 g of leaf litter). More than 98.5% of ticks overwintering in the field in balsam fir died (69/70), but only 57.1% (40/70) died in maple/oak leaf litter (G-test = 38.2, *p* < 0.0001). Balsam fir killed both male (N = 34/34 dead) and female (N = 35/36 dead) ticks. Ticks died in balsam fir leaf litter whether the tubes were placed in a forest environment (N = 31/32 dead), or in dune grass (N = 38/38 dead). Adult female ticks also died in balsam fir leaf litter when held in incubators simulating winter conditions for 12 weeks. During the simulated winter (Fig. S1), 100% of ticks in balsam fir died (48/48), while only 20.8% (10/48) of ticks in maple/oak died (G-test = 72.9, *p* < 0.0001). Balsam fir needles killed ticks infected with *B. burgdorferi* as well as non-infected ticks (Field–100% infected died (8/8) and 98.4% uninfected died (61/62); Incubator–100% of infected (33/33) and 100% non-infected (15/15) died). The nymphal stage was also killed by balsam fir needles. Lab-reared *I. scapularis* adult females and nymphs were obtained from the National Tick Rearing Facility in Oklahoma, USA and were placed in individual tubes. The incubator was set at 0 °C ± 1 °C for 10 weeks. As expected, all adult females in balsam fir (5/5) died during the simulated winter, while 60% survived in maple/oak (3/5), demonstrating that both field-collected and lab-reared ticks are susceptible to balsam fir needles. Overwintering nymphs died in balsam fir needles (0/26 survived in balsam fir, 22/24 survived in maple/oak, Fisher Exact Test, *p* < 0.0001).

These results could be due to differences in the insulating properties of the two leaf litters. Maple/oak leaf litter did have better insulating properties compared with balsam fir, but the differences were small. The average temperature inside both leaf litters varied between each other by only 0.5° ± 0.4°C, averaged across 3 incubators with different winter temperature regimes (see Supplementary Information). Differences in relative humidity between the two leaf litters were larger, with maple/oak having higher relative humidity on average (5.5% RH ± 0.4% higher) than balsam fir leaf litter. Daily fluctuations in relative humidity were also larger within the balsam fir leaf litter (e.g., daily range min–max RH, balsam fir = 6.3% ± 9.8%, maple/oak = 4.1% ± 5.6%).

**Figure 1:**
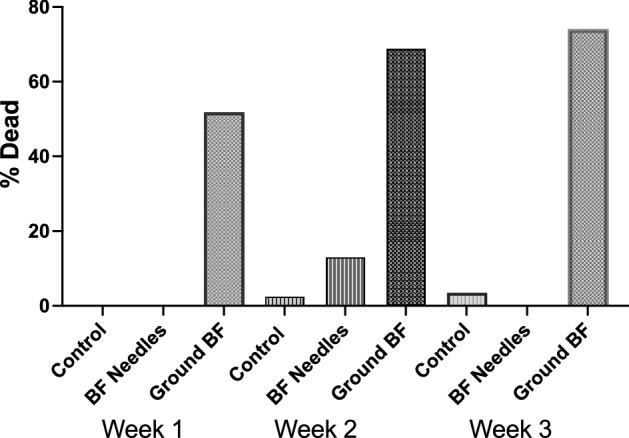
Effect of intact and ground balsam fir needles on the survival of adult female *Ixodes scapularis* exposed to 0–4 °C. N = 27 ticks/group for Week 1 and Week 3. For Week 2, N = 36 for controls and N = 45 for intact or ground balsam fir needles (BF Needles—intact balsam fir needles, Ground BF—ground balsam fir needles).

To avoid the possibility that the effect we observed was due to the small differences in leaf litter insulating properties, we used a vermiculite/sand mixture or cotton ball as the substrate at the bottom of the tubes in subsequent experiments. Grinding balsam fir needles increased their acaricidal effect in incubators simulating winter conditions (Fig. 1; Chi-square (2) = 149, *p* < 0.0001, difference across groups. Linear trend with time for ground needles, Chi-square (1) = (3.26), *p* = 0.07). The acaricidal effect of ground needles was not immediate but required about 3 weeks to kill 75% of the ticks (Fig. 1). Ticks housed with intact needles were still alive 3 weeks later, suggesting that balsam fir needles require several weeks to kill ticks. However, although ticks were not killed outright by balsam fir needles, after 2 week exposure, ticks suffered from slower righting reflexes than controls in all 3 winter incubation temperatures (Fig. S2, 1-way ANOVA, F(5, 127) = 44.35, *p* < 0.0001, post hoc comparisons using a Tukey test, 0 °C, *p* < 0.0001; 4 °C, *p* < 0.0001, Fluctuating − 2° to + 2° C, *p* < 0.0001, no differences across the 3 controls, F(3, 58) = 0.80, *p* = 0.45).

### Acaricidal compounds in balsam fir needles

To determine whether balsam fir needles contained compounds that kill ticks, we fractionated ground balsam fir needles (1 g balsam fir needles ground with 10 mL double distilled water). The slurry was filtered using Whatman #4 filters. The green filtrate smelt strongly of balsam fir. This material was added to 10 K MWCO centrifuge filters. The clear aqueous fraction at the bottom of the centrifuge tube had no smell, while the material that did not pass through the filter was a green colour and retained the smell of balsam fir. Three-hundred (300) µL of either the clear aqueous fraction or 300 µL of the green residue fraction was added to a sand/vermiculite mix at the bottom of a tube. The tubes, containing a single lab-reared adult female, were placed in an incubator set at 0°C for 4 weeks; 80.9% of ticks (N = 21) died when exposed to the green filtrate, while fewer than 15% of ticks died when exposed to the clear filtrate (14.3%, N = 21) or water (300 µL of water added, 11.1%, N = 18; G-test (2) = 26.6, *p* < 0001). Based on these results, we explored the possibility that an essential oil may be involved in balsam fir’s acaricidal effect.

**Figure 2:**
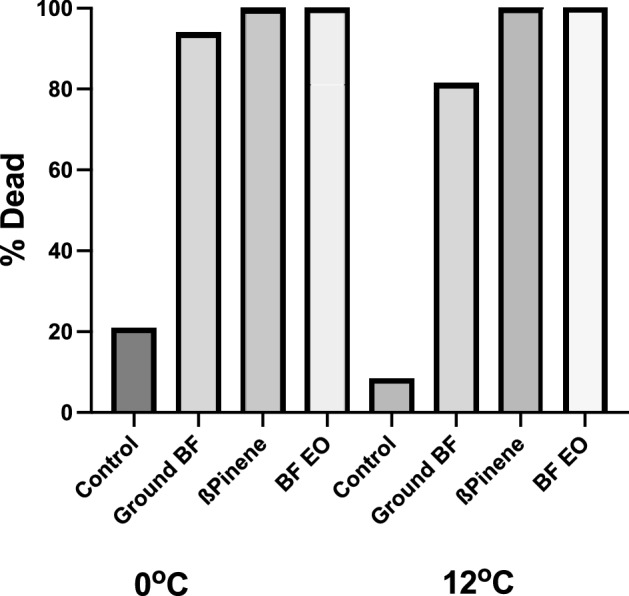
Ground balsam fir needles, ß-pinene and balsam fir essential oil kill ticks at two different temperatures (0 °C and 12 °C). Bars represent the percentage of dead ticks after 4 weeks. BF = Balsam Fir, BF EO = Balsam Fir Essential Oils. Control N = 24/incubator, Ground BF N = 16/incubator, ß-pinene N = 24/incubator, BF EO N = 32/incubator.

First, we determined the essential oil composition of our locally collected balsam fir using gas chromatography mass spectrometry after extracting the oil by hydro-distillation. The essential oil composition of fresh balsam fir needles showed an abundance of ß-pinene (Table 1; Fig. S3), as was found by others^30,31^. To mimic the amount of balsam fir or ß-pinene essential oil that would be found in 2 g of balsam fir needles (based on data from Poaty et al.^31^), we added 2 mL of 1.75% (v/v) of balsam fir essential oil or 0.65% (v/v) of ß-pinene in 1% Tween 80 (v/v) in double distilled water to a cotton ball at the bottom of each tube. One lab-reared adult female tick was placed in each tube, and the tubes were placed in incubators to simulate overwintering conditions for 3 weeks. Ticks exposed to ground balsam fir needles (positive control), ß-pinene and balsam fir essential oil died, while ticks in control tubes (1% v/v Tween 80) survived (Fig. 2, Planned comparisons for contingency tables^32^; Control significantly different than other groups, 0 °C: Z = 8.1; *p* < 0.0001; Overall Chi-square(3) = 66.3, *p* < 0.0001. 12 °C: Control significantly different than other groups (Z = 8.5; *p* < 0.0001). Overall Chi-square (3) = 73.8, *p* < 0.0001).

**Table 1:** Compounds identified in Balsam fir needles by GC–MS.

	Compound	ID	RT (min)	% Analyte in Total oil ± SE
1	Santen	NIST	7.131	1.50 ± 0.11
2	Tricyclene	NIST	8.368	1.11 ± 0.04
3	3-Methyl-4-heptanol	NIST	8.495	2.69 ± 0.02
4	α-pinene	S	8.726	7.09 ± 0.18
5	Camphene	S	9.205	7.98 ± 0.15
6	4-hepten-2-one	NIST	9.399	1.53 ± 0.12
7	8-hydroxylinalool	NIST	9.732	9.34 ± 0.11
8	β-pinene	S	10.017	22.5 ± 0.44
9	β-myrcene	S	10.377	0.59 ± 0.03
10	3-carene	S	10.897	10.3 ± 0.15
11	Limonene	S	11.455	3.44 ± 0.10
12	β-phellandrene	NIST	11.482	5.38 ± 0.06
13	Isoborneol	NIST	14.946	0.67 ± 0.06
14	Borneol acetate	S	17.342	18.0 ± 0.23
15	Caryophyllene	S	20.044	2.18 ± 0.16
16	Humulene	S	20.690	1.12 ± 0.15
17	β-bisabolene	NIST	21.560	1.22 ± 0.12
	Unknowns	-	-	3.6 ± 0.2

RT= Retention Time, ID= Identification Reference Source, NIST=National Institute of Standard Technology, S=Chemical standard.

Essential oils are known for their volatility (e.g. balsam fir^33^). In the incubator trials described above, the tubes were sealed with parafilm to maintain high humidity. However, this method also concentrates released volatiles, increasing the concentration in the headspace. We thus tested acaricidal effect of the essential oils whether the tubes were left open (i.e. entrance covered only by a fine mesh), mimicking an outdoor environment in which volatiles could dissipate. High humidity was maintained in the tubes, and the cotton remained damp throughout the 4-week trial. Mortality was greater under closed tube conditions and depended on essential oil concentration (Fig. S4; Chi-square test. Significant difference between closed and open tubes, Chi-square (1) = 25.7, *p* < 0.00001, Significant difference across concentrations, Chi-square (2) = 35.1, *p* < 0.0001). We also found that balsam fir essential oil was more toxic to ticks than ß-pinene (Chi-square test. Significant difference between balsam fir essential oil (N = 112) and ß-pinene (N = 48), Chi-square (1) = 15.0, *p* < 0.0001, significant difference across concentrations, Chi-square (2) = 35.2, *p* < 0.0001).

**Figure 3:**
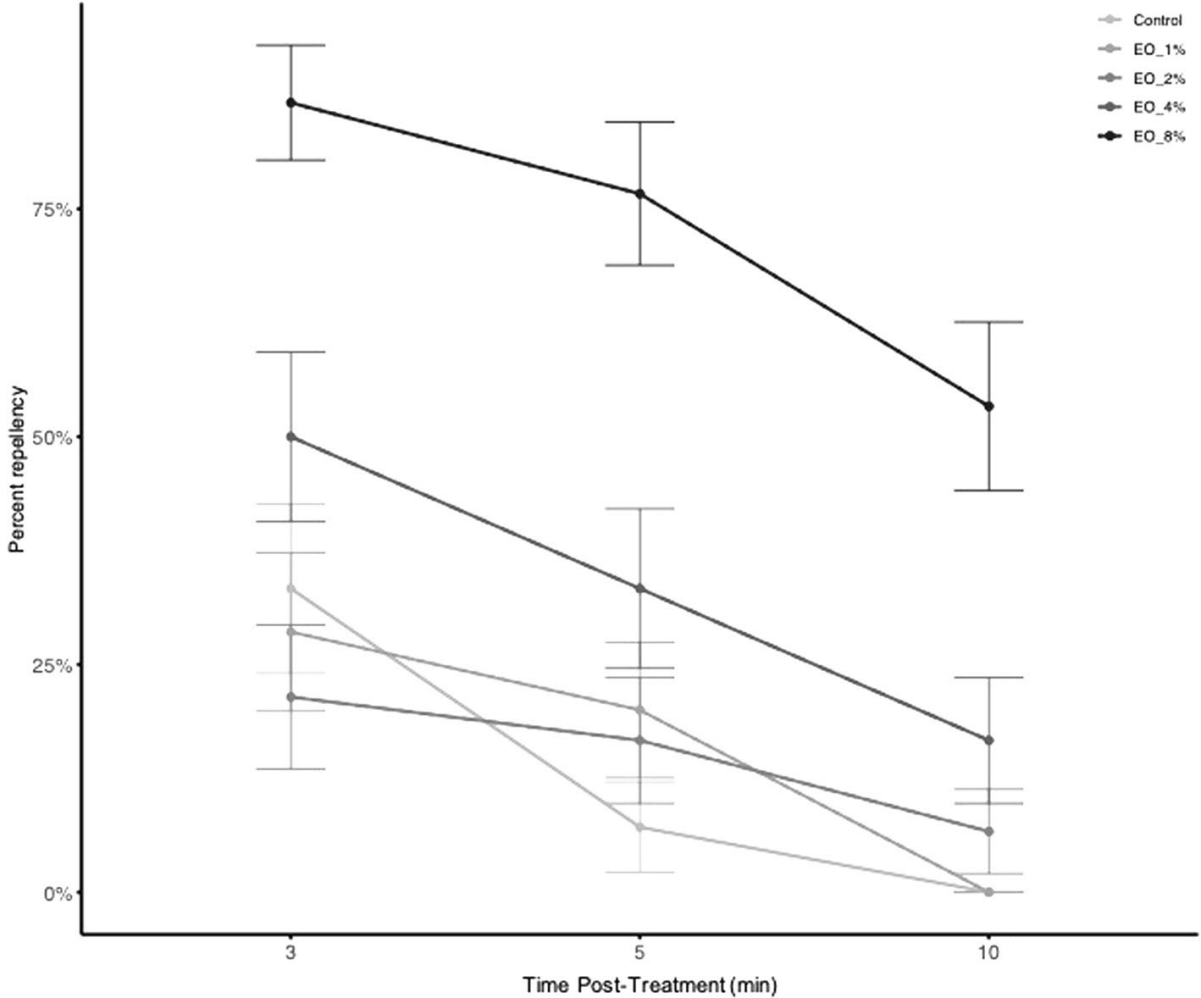
Mean repellency (%) over time of *Ixodes scapularis* nymphs exposed to balsam fir essential oil (EO) at different concentrations (1–8% v/v). N = 30 per treatment.

Given the acaricidal effects of balsam fir, we also tested whether balsam fir was a repellent using horizontal bioassays according to Faraone et al.^34^. Balsam fir essential oil was repellent for up to 10 min at room temperature, and the effect was concentration dependent (Fig. 3, F4,436 = 34.65, *p* < 0.001, N = 30/group). The lowest concentration that exerted a significant repellent effect for up to 10 min was 4% v/v (z = 3.087, *p* = 0.002); 86.7% of the tested ticks were repelled when exposed to 8% v/v balsam fir essential oil after 3 min of exposure (Table S1). The repellent effect decreased over time (Fig. 3).

### Effect of temperature on the acaricidal activity of balsam fir essential oil

**Figure 4:**
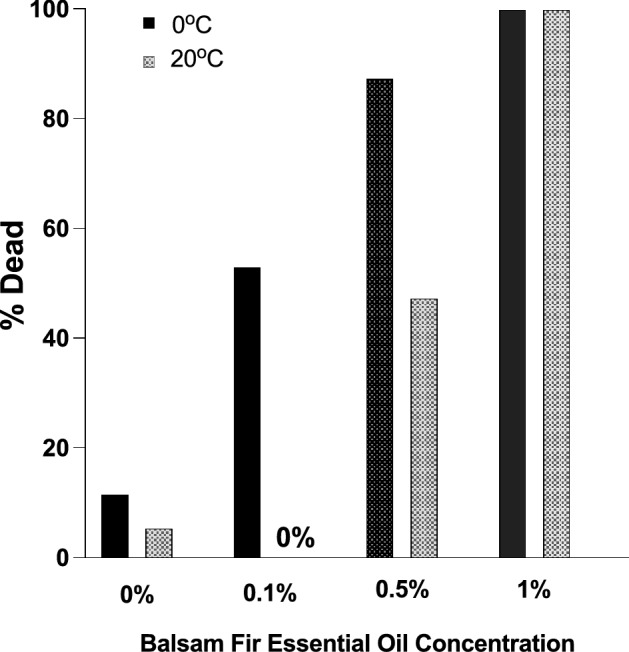
Balsam fir essential oil killed ticks at a lower concentration at 2 °C than at 20 °C. The black bars represent the mortality at 2 °C while the grey bars represent mortality at 20 °C. N = 32 for each bar except N = 36 at 0%.

To test whether low temperature enhanced the acaricidal effect of balsam fir essential oil, we placed adult female ticks in individual tubes in one of two incubators (2 °C and 20 °C). At 20 °C, a higher concentration of balsam fir essential oil was required to kill ticks compared to 2 °C (Fig. 4; Significant temperature effect, Pearson Chi-square test (1) = 19.64, *p* < 0.0001; Significant effect of essential oil concentration, Pearson Chi-square (3) = 128.7, *p* < 0.0001).

## Discussion

Balsam fir needles killed overwintering ticks. The essential oil extracted from these needles was also acaricidal. The main compound within balsam fir essential oil, the monoterpene ß-pinene, killed ticks too, although less effectively than the whole balsam fir essential oil. Balsam fir needles required several weeks to kill ticks, while the essential oil killed ticks in days at cold temperatures. Room temperature (i.e., 20 °C) reduced the tick-killing effectiveness of the essential oil (Fig. 4). Lower temperatures may have decreased the loss of the volatile compounds responsible for the tick-killing. By decreasing the loss of these compounds, the duration of the tick’s exposure to the essential oil could have been increased.

Low temperatures may aid in killing ticks for reasons other than reducing the loss of volatile substances. Low temperatures slow enzyme activity^15^ and could reduce the rate of detoxification, allowing toxic compounds to accumulate. In addition, detoxification pathways may be compromised by a shift in resources towards overwintering survival. In some insects, detoxification is impaired during the activation of other physiological systems (e.g., immune responses) due to competition for limited resources^35^. Exposure to toxic compounds may force an over-investment in detoxification; such an over-investment may impair overwintering survival^36^. Some insects experience oxidative stress during exposure to low temperatures^37^. If ticks do the same, then low temperatures could compound the toxic effects of balsam fir. Additionally, the decline in mobility at low temperatures^38,39^ may limit the ability of *Ixodes* ticks to move away from toxic leaf litter. Although this was not a serious issue in this study, as ticks were confined to tubes, it would reduce the ability of ticks to exit toxic leaf litter in the field. Even though ticks are capable of some movement in the winter^39^, forcing ticks to expend energy in locomotion during the winter to avoid toxic leaf litter could reduce overwintering survival during this non-feeding state. Not only will ticks be forced to consume scarce reserves, but they will also be more vulnerable to predation, freezing, and desiccation while searching for a new patch of leaf litter. In addition, ticks exposed to balsam fir needles showed evidence of toxicity even after a short exposure (i.e., a slower righting reflex). These sublethal effects could produce a reduction in motor abilities, leaving ticks unable to find hosts. Thus, even if ticks are able to leave balsam fir leaf litter, shorter exposures may nonetheless impact tick populations.

This study used both locally collected *I. scapularis* ticks, as well as lab-reared *I. scapularis* ticks from the facility at the University of Oklahoma. These two populations of ticks could have both physiological and behavioural differences^40^. Regardless, balsam fir needles reduced successful overwintering in ticks from both populations, pointing to the robustness of the result. However, the studies exploring the effects of balsam fir essential oil were performed on the lab-reared ticks only. Future studies should expand an examination of the temperature/essential oil interactions on other populations of *I. scapularis* ticks, as well as other tick species to establish the generalizability of the result. In addition to ß-pinene, other components are likely to play a role in the acaricidal effect of balsam fir essential oil. Synergistic interactions between components could explain why balsam fir essential oil is more effective than ß-pinene alone. The combination of different essential oil components can enhance their overall efficacy^41^, and balsam fir contains a number of monoterpenes and other compounds (Table 1). Two of the main monoterpenes found in our locally collected balsam fir needles, α-pinene and ß-pinene (Table 1), are known to be lethal to some insects^42^. β-pinene has also been reported to repel *Amblyomma americanum* and *Rhipicephalus appendiculatus* ticks, although it has not previously been identified as an acaricide^41^. In fact, α- and β -pinene were not effective at killing *I. scapularis* nymphs at any concentration in a previous study^43^ that examined their acaricidal effect at 21 °C and for 24 h. α -pinene and β -pinene may be lethal only at colder temperatures. The effect of plant compounds on ticks are often tested at temperatures greater than 20 °C (e.g., incubating for 15 days at 27^o^C^44^), which could explain why some plant compounds may be overlooked.

Balsam fir essential oil showed a significant repellent effect at room temperature, but it required a relatively high concentration (i.e., 8% v/v) in the horizontal assay test. Other plant essential oils can repel ticks (such as *I. ricinus*) at lower concentrations (e.g., lavender oil, < 1% w/w^45^). The repellency effect is significantly reduced after only 10 min, indicating the high volatility of the essential oil (Fig. 3). This property would reduce its usefulness as a repellant as it would require frequent re-application. These results demonstrate the low persistence of balsam fir essential oil at room temperature.

Our findings suggest that balsam fir needles, and their essential oil, have development potential as an acaricide. Balsam fir essential oil has minimal toxicity to terrestrial vertebrates at the concentrations used in this study^46^. A balsam fir-derived product could provide environmentally-friendly control, especially when used during the winter when ticks are most sensitive to it. Moreover, winter application of an acaricide could offer additional ecological advantages. By late fall fewer non-target organisms are likely to be exposed^47^. Therefore, later applications should reduce potential negative impacts on pollinators^48^. Late application would also reduce the phytotoxicity that may occur with some essential oils^48^. However, the compounds in plants that are deadly to ticks probably evolved to defend plants against insect herbivores, not blood-sucking arthropods^2^. They have effects on ticks because of similarities in the physiology of insects and ticks^2^. Therefore, any development of balsam fir, or other plant-based product targeting the over-wintering period, should examine its effects on arthropods that over-winter in the ground, especially on soil organisms that help control ticks^49^.

Whether other plants can decrease tick overwintering survival should also be explored. In previous studies that showed high overwintering success in *I. scapularis*, the leaf litter was from deciduous trees^14,22,50^. Ticks appear to have better survival and are more abundant in deciduous forests during their active period (i.e., spring, summer and fall) than in coniferous forests^51–54^, although not all studies find these trends^17,27^. Nevertheless, our results suggest that some species of conifers may be a potential source of anti-tick compounds. We hypothesize it will be plants like balsam fir, with which *I. scapularis* ticks historically have had little experience. For example, few *I. scapularis* ticks are found under eastern hemlock (*Tsuga canadensis*)^53^, suggesting another possible northern conifer with acaricidal properties.

Although low humidity can be lethal to *I.* scapularis^27,55^, it is unlikely that the decline in tick survival in balsam fir leaf litter was because it was a poor insulator. Lindsay et al.^56^ found little difference in temperature or vapour pressure deficit between white pine and maple forest leaf litter over the winter. Brunner^14^ found no differences in the overwintering survival of *I. scapularis* nymphs between two sites that differed in relative humidity by about 20% (one site had 40% RH, the other had 20% RH). This result suggests that the small differences we observed in humidity between the two leaf litters in this study are unlikely to explain the very large difference in mortality.

Many people become infected with Lyme disease in their own yard; a safe, efficient acaricide could play a role in reducing the exposure to tick bites and to the spread of these infections^4,5^. Even if a vaccine for Lyme disease becomes available, *I. scapularis* can vector other diseases^57^, and tick control will be necessary to prevent them. However, how well reducing the number of ticks around residences will reduce the transmission of tick-borne diseases remains uncertain^58,59^. For example, reducing tick numbers near homes reduced tick-borne diseases in pets, but not in people^59^. Nevertheless, our results show that a single application of balsam fir needles, or a balsam fir-based product, could be sufficient to kill overwintering ticks. Despite winter rain and periodic snow melts, a single patch of balsam fir needles was able to kill ticks in our open tube field tests. The next step will be demonstrating whether balsam fir needles can kill overwintering ticks under real world conditions. For example, if prolonged contact between tick and balsam fir is required to kill ticks, ticks that move to a less toxic overwintering site (e.g., horizontally to a needle-free zone, or deeper into the leaf litter below the balsam fir needles), could evade control.

Other studies have noted that applications of acaricides prior to re-activation of ticks in the spring can significantly reduce the numbers of early season ticks^11^, suggesting that reducing overwintering survival will reduce numbers in the spring^60^. For example, Rand et al.^61^ found that a low toxicity acaricide (i.e., IC2™, formulation based on rosemary essential oil) sprayed in October substantially reduced the number of *I. scapularis* ticks in the following spring. However, the same low toxicity control agent (i.e., IC2™) applied in June had little lasting effect^10^. Possibly the application of IC2™ under cold weather conditions (i.e., late October in Maine, US) enhanced its efficacy^61^. Cold temperatures may be an important ally of essential oil-based products.

## Methods

### Ticks

Studies used unfed nymph and adult *I. scapularis* ticks. Ticks were either locally collected in Nova Scotia (i.e. Halifax Regional Municipality, Lunenburg County or Port Joli, Nova Scotia, Canada), or were purchased from the National Tick Research and Education Resource, housed at Oklahoma State University (OSU) (Stillwater OK, USA). Ticks from OSU were used in the repellent studies and studies examining the effect of temperature on the acaricidal ability of balsam fir essential oil. Studies were approved by the University Committee on Laboratory Animals at Dalhousie University (protocol number: I18-06). Research on plants in this study complies with the Convention on Biological Diversity^62^ and the Convention on the Trade in Endangered Species of Wild Fauna and Flora^63^.

### Effects of balsam fir needles on overwintering I. scapularis in the field

Field work was conducted at the Harrison Lewis Coastal Discovery Centre (HLC), Port L’Hebert, NS (HLC43°49′04.2″ N 64°53′40.4″ W). *Ixodes scapularis* were collected by dragging^54^ from the HLC and Thomas Raddall Provincial Park, located adjacent to the HLC. Following collection, 70 ticks were placed in individual microcosms using a design modified from Burtis^64^. The microcosm was an open plastic tube (15.24 cm in length × 1.27 cm in width), with mesh glued to each end, secured by a plastic cap. The plastic caps had a central hole that allowed rain and snow to enter in the top and connected the soil with the open bottom of the tube. The tubes contained sand and vermiculite (75% w/w sand and 25% w/w vermiculite) to a depth of 5 cm in the tube. The tubes were placed 5 cm into the ground. On top of the sand/vermiculite was 1 cm of either a) cut oak (Red oak (*Quercus rubra* L.) and maple (Red maple (*Acer rubrum* L.) leaves or b) 1 cm of cut balsam fir (*Abies balsamea* L., Mill.) needles. Plants were identified by SAA or LF using a key from the Government of Nova Scotia^65^. A single tick was placed into each microcosm and then the microcosm was placed under a coarse netting (NuVue® (78.7 cm L × 78.7 cm W × 83.8 cm H). A data logger (HOBO MX2301A) was placed in each enclosure with a probe 5 cm underground between the microcosms; the logger was covered with a solar radiation shield (Onset RS3-B) and the logger automatically recorded temperature and relative humidity every hour. Tick vials and data loggers were placed on October 25, 2018 and retrieved on February 8, 2019. Ticks were placed in either dune grass (N = 38) or in a mixed maple/spruce stand of trees (N = 32) behind the dune grass.

Ticks were recovered by carefully sieving the tubes. For all the experiments described in this paper, mortality was determined by allowing ticks to return to room temperature (approximately 22 °C) for 24 h. Ticks that were immobile after both a gentle exhalation by the experimenter (AEN) and probing with a paint brush, were declared dead. Both dead and living ticks were tested for the presence of *B. burgdorferi* DNA using qPCR (see Supplementary Information).

### Effects of balsam fir needles on I. scapularis overwintering in incubators

Ticks were collected in Burnside (Nova Scotia, Canada) and maintained at 4°C for 1 to 6 weeks prior to the study. Ticks were placed in glass tubes of the same size (15.24 cm in length × 1.27 cm in width) as was used in the field study, and had the same contents, but these were sealed at the bottom and capped at the top with parafilm. They were placed in one of 3 incubators simulating winter conditions (see Supplementary Information for details, Fig. S1). Ticks were placed in incubators in late fall and removed 12 weeks later. Tick mortality was recorded, and then ticks were tested for *B. burgdorferi* (Supplementary Information).

### Essential oil extraction from balsam fir needles

Fresh balsam fir stems and leaves were collected in Port Joli and Lunenburg County (Nova Scotia, Canada). Needles were manually removed from the branches and ground using an electric coffee grinder (Black + Decker). Plant material was stored at − 20 °C until required. Essential oil extraction through hydro-distillation was performed using a Clevenger-type apparatus as previously described (Wang et al., in review). Hexane and deionized water were used as the solvent for the balsam fir extractions. Anhydrous sodium sulfate was used for drying balsam fir oil after extraction. Essential oil was stored at − 20 °C.

### GC–MS analysis and characterization of balsam fir essential oil

Essential oil samples and chemical standards were analyzed via gas chromatography-mass spectrometry (GC–MS) to determine composition and main constituents. Essential oil was diluted in hexane at a concentration of 50 ng/μl. Samples were analyzed using a Scion 456 Gas Chromatograph-Single Quad (GC–MS; SCION Instruments UK Ltd., Livingston, UK). A non-polar capillary column Rxi®-5silms (30 m × 0.25 mm, film thickness 0.25 mm; Restek Corporation, State College, PA, USA) linked to a Bruker mass spectrometer (Bruker Daltonics Ltd., Coventry, UK) was used for analysis. One μl was injected at 250 °C by using a Gerstel Maestro Autosampler (Gerstel, Mülheim an der Ruhr, Germany). The split ratio for chemical standards was set up as 1:100. The oven temperature was set up at 50 °C for 5 min, then increased to 220 °C at 7 °C/minute, and then increased up to 280 °C at 30 °C/minute. Both the injector and transfer line were maintained at 250 °C. Helium was used as a carrier gas at 1 mL/min rate flow. Compounds were identified using analytical standards and NIST Mass Spectral Search Program for the NIST/EPA/NIH Mass Spectral Library Version 2.0 g 2011 (Scion Instruments UK Ltd., Livingston, West Lothian, UK).

### Test of the acaricidal activity of balsam fir essential oil

Lab-reared ticks from OSU were placed singly in tubes as described for the incubator study. We added 2 mL of 1.75% (v/v) of balsam fir essential oil in 1% (v/v) Tween 80 (Sigma-Aldrich, Saint Louis, MO, US) to each tube (N = 32/incubator) to mimic the amount of balsam fir essential oil found in 2 g of balsam fir needles^31^. We also added 2 mL of 0.65% (v/v) β-pinene in 1% (v/v) Tween 80 (N = 24/incubator) to mimic the amount of β -pinene found in 2 g of balsam fir needles^31^. Control tubes received 2 mL of 1% (v/v) Tween 80 (N = 24/incubator). Tubes were placed in incubators set at 0 °C and 12 °C for 4 weeks.

### Balsam fir oil horizontal repellency bioassay using nymphs

Lab-reared ticks from OSU nymphs were stored in sealed plastic containers lined with moist Kimwipe® in the fridge at 4°C in dark conditions prior to trials. Balsam fir essential oil was diluted to 4% (v/v) or 8% (v/v) in hexane, with hexane acting as the control. Each concentration was tested against five nymphs for a total of 6 replications (N = 30). The horizontal repellency bioassay was performed as described in Faraone et al.^34^ with some modifications. Briefly, the experimental arena was composed of three circles: the dropping zone (i.e. inner circle), treated circle, and outer circle. 320 $\mu$ L of either the 4%, 8%, or control solution was added to the treated circle. Five active nymphs were transferred to the dropping zone by using a fine paintbrush. The location of each tick was recorded at 3, 5, and 10 min. Active ticks, which exited the dropping zone, but did not cross the treated circle, were recorded as repelled. Active ticks that crossed into the treated circle or into the outer untreated circle were recorded as non-repelled.

### Effect of temperature on the effect of balsam fir essential oil

Adult lab-reared ticks from OSU were placed singly in tubes as previously described above. Based on the results of the previous experiments, we used the following balsam fir essential oil concentrations: 0.1%, 0.5% and 1% (v/v) (N = 32; 16 males and 16 females/concentration) in 1% Tween 80 for each temperature. We added 2 mL of each concentration to tubes with cotton as described above. Control tubes received 2 mL of 1% Tween 80 (N = 36/temperature; 18 males and 18 females). Tubes were placed in incubators set at 2 °C or 20 °C for 2 weeks. Mortality was assessed. The results for both sexes were pooled because they did not differ significantly (Pearson Chi-Square (1) = 2.184, *p* = 0.139).

### Statistical analysis

All data were tested for the assumptions of the statistical test used. Statistical analyses for contingency tests were performed on SPSS (ver. 26). Other tests were performed using Prism (GraphPad, Version 9.20) or RStudio Version 1.1.453 (RStudio Team 2009–2018). When multiple statistical tests were performed on the same data set, the alpha criterion was adjusted using a Bonferroni correction. Most of the data were categorical and were analyzed using Pearson Chi-square with Bonferroni correction. For the repellency tests, efficacy was analyzed with generalized linear mixed effect regression (glmer) with a binomial link to model the logit of tick repelled, followed by simultaneous tests for general linear hypotheses and multiple comparisons of means (Tukey’s test), using the emmeans package.

## Supplementary Information

Supplementary Information.

## Data Availability

Data are available on Mendeley (https://data.mendeley.com//datasets/gmgg47rcb5/1).
