# Supplementary material for: Balsam fir (Abies balsamea) needles and their essential oil kill overwintering ticks (Ixodes scapularis) at cold temperatures
Source: Sci Rep. 2022 Jul 29;12:12999. doi: 10.1038/s41598-022-15164-z (PMC9338056; doi:10.1038/s41598-022-15164-z)
Supplement: Supplementary file 1 — Supplementary Information. [file 41598_2022_15164_MOESM1_ESM.docx]

**Balsam fir (*Abies balsamea*) needles and their essential oil kill overwintering ticks (*Ixodes scapularis*) at cold temperatures**

Shelley A. Adamo^1*^, Amal El Nabbout^1^, Laura V. Ferguson^1,2^, Jeffrey S. Zbarsky^1^, Nicoletta Faraone^3^

1. Dept. Psychology and Neuroscience, Dalhousie University
2. Dept. Biology, Acadia University
3. Dept. Chemistry, Acadia University

**Supplementary Information**

*Winter Incubator Temperatures*

Incubator temperatures were designed to mimic the historical average winter temperatures (i.e. 0^o^C for several weeks), predicted winter temperatures in 2080 (2^o^C to 8^o^C), and a winter with enhanced temperature fluctuations, as is predicted to occur with climate change in Nova Scotia [1]. The incubators (Fisherbrand, models 3724 and 97990E) were set to a short day-length period (10 h light: 14 h dark) to mimic winter conditions. Fig. S1 shows the temperature regime for each incubator.

*Determination of tick infection status*

Ticks were identified using standard keys [2]. DNA was extracted from *I. scapularis* following the Public Health Agency of Canada (PHAC) protocol, adopted from Courtney et al. [3] by using a Qiagen QIAamp**^©^** DNA Mini Kit with an extra bead tube step (MN Bead Tubes Type D (Macherey-Nagel)) to enhance the DNA extraction process. MN Bead Tubes Type D (Macherey-Nagel) were filled with 200 μl of Qiagen ATL buffer. Individual ticks were crushed thoroughly using a Bel-Art^®^ Disposable Pestle. Bead tubes were vortexed for 30 minutes. 20 μl of Qiagen Proteinase K was then pipetted into each bead tube. Bead tubes were incubated at 56°C for 60-90 minutes. 200 μl of Qiagen AL buffer was added to each bead tube and tubes were vortexed for 15 seconds and then incubated at 70°C for 10 minutes. Finally, 200 μl of 100% ethanol was pipetted into each bead tube and then tubes were vortexed for 15 seconds. DNA extraction followed QIAamp**^©^** DNA mini kit instructions.

DNA was then screened for evidence of *B. burgdorferi* infection using a multiplex real-time PCR targeting the 23S rRNA of *B. burgdorferi*. *B. burgdorferi* infection was then confirmed in positive samples using primers targeting the ospA gene. Each sample was tested in triplicates and all plates included: a no template control (5 Microliters of milliQ water), a 25 cq value positive control, and a 30 cq value positive control. The thermocycling conditions occurred as follows: activation of enzyme AmpErase at 50°C for 2 minutes, denaturation of AmpErase and activation of AmpliTaq Gold^®^ Polymerase at 95°C for 10 minutes, followed by 40 cycles of amplification at 95°C for 15 seconds and annealing at 60°C for 1 minute.

The integrity of DNA samples was assessed using gel electrophoresis using a Gel Doc™ XR+ Digital Gel Imaging System^®^ from Bio-Rad^®^ [4]. DNA quantity was determined using a Qubit fluorometer.

We found that *B. burgdorferi* DNA could be detected in ticks several weeks after their death under winter conditions (El Nabbout, in preparation).

Primer sequences used in *B. burgdorferi* identification

| **Primer or Probe** | **Sequence 5’ to 3’** |
| --- | --- |
| **Bb 23S (forward)** | CGAGTCTTAAAAGGGCGATTTAGT |
| **Bb 23S (reverse)** | GCTTCAGCCTGGCCATAAATAG |
| **Bb 23S Probe FAM-TAMRA** | AGATGTGGTAGACCCGAAGCCGAGTG |
| ***ospA* (forward)** | CTGGGGAAGTTTCAGTTGAAC |
| ***ospA* (reverse)** | TTGGTGCCATTTGAGTCGTA |
| ***ospA* Probe FAM-BBQ** | CTGCAGCTTGGAATTCAGGCACTT |

*Determining the insulating properties of maple/oak versus balsam fir leaf litter*

To determine whether the difference in mortality may be due to differences in insulation properties between maple/oak leaf litter and balsam fir, we filled 100 mL beakers with maple/oak leaf litter or balsam fir needles. The HOBO logger probe was placed inside the leaf litter, in the center of the beaker. Each of the 3 incubators (see Fig. S1 for temperature regime) received 1 balsam fir beaker and 1 maple/oak beaker. The temperature and relative humidity within the leaf litter were recorded.

*Righting reflex after exposure to balsam fir needles.*

Individual, locally-collected *I. scapularis* adult female ticks were placed in microcosm tubes inside a 0°C incubator, 4°C incubator, or fluctuating incubator (0°C to 4°C) for two weeks. Ticks were randomly assigned to one of two groups: control group (sand and vermiculite in the tube along with 2 mL of water), and balsam fir needle group (sand, vermiculite, balsam fir needles, and 2 mL of water). At the 14-day mark, individual ticks from each group were removed from each incubator and were placed on their dorsal surface in a petri dish at 25°C. The time required for the tick to right itself was recorded.

*Exploration of possible balsam fir acaricidal components.*

Lab-reared adult female ticks were placed singly in tubes as described above for the incubator studies, except that a cotton ball was placed at the bottom of the tube to provide an artificial substrate. Balsam fir needles were ground with a mortar and pestle with double distilled water (1g needles: 10 mL water). To test whether ground balsam fir needles were also acaricidal, ticks were placed in tubes with 2mL of ground balsam fir, balsam fir needles (approx. 2 g/tube) or 2 mL of water. Tubes were placed in incubators with winter temperatures (0^o^C, 4^o^C or fluctuating between -2^o^C to +2^o^C). There was no significant difference in mortality across incubators (Chi squared (2)=1.83, p=0.40, N=96/incubator) and results between incubators were pooled. Every week for the next 3 weeks a subset of tubes (week 1, 27 tubes/group; week 2, 36 control tubes and 45 tubes balsam fir needles and ground balsam fir, week 3, 27 tubes/group) were removed and the number of alive vs dead ticks was recorded. Ticks removed for testing were not returned to the incubator.

Ground balsam fir was filtered using a Whatman #4 filter papers. A green filtrate, with the typical odour of balsam fir, was then placed in VWR centrifuge filters (MWCO 10 K) and spun at 12,000 g for 10 min at 4^o^C; 300 µL of the clear filtrate or 300 µL of the green residue was added to the tubes prior to placing them in the incubator set at 0^o^C or 12^o^C for 4 weeks.

*Closed versus Open Tube Study*

To test whether an open tube would reduce the effectiveness of essential oils, we added 1%, 0.5%, 0.1% and 0.05% (v/v) β-pinene in 1% (v/v) Tween 80, or 1% (v/v) Tween 80 alone as a control to both closed and open tubes. One adult lab-reared female was added to each tube. Closed tubes were sealed with parafilm as done previously, while open tubes were covered with a mesh. Tubes were placed in an incubator set at 0^o^C for 3 weeks. The number of alive and dead ticks was recorded (Fig. S4)

*Balsam fir essential oil versus β-Pinene essential oil*

To test whether β-pinene was as toxic to ticks as was balsam fir essential oil, lab-reared adult female ticks were placed in open tubes as described above with 2%, 1% or 0.5% (v/v) of one of either Balsam fir essential oil or β-pinene essential oil in 1% (v/v) Tween 80. 1% (v/v) Tween 80 (control) was used as the control. Tubes were placed in an incubator for 4 weeks at 0^o^C. The number of alive and dead ticks was recorded.

**References**

[1] **Bush, E. and Lemmen, D. S.** (2019). Canada’s Changing Climate Report. Ottawa, ON: Government of Canada.

[2] **Keirans, J. E. and Litwack, T. R.** (1989). Pictorial key to the adults of hard ticks, family Ixodidae (Ixodida: Ixodoidea), east of the Mississippi River. *Journal of Medical Entomology* **26**, 435-448.

[3] **Wills, M. K., Kirby, A. M. and Lloyd, V. K.** (2018). Detecting the Lyme Disease spirochete, *Borrelia burgdorferi.* . *Journal of Visualized Experiments (JoVE)* **132**.

[4] **Courtney, J. W., Kostelnik, L. M., Zeidner, N. S. and Massung, R. F.** (2004). Multiplex real-time PCR for detection of *Anaplasma phagocytophilum* and *Borrelia burgdorferi.* *Journal of Clinical Microbiology* **42**, 3164-3168.

**Supplementary Tables**

**Table S1.** Mean percentage (±SEM) of ticks repelled by balsam fir essential oil at different concentrations (% v/v). Repellency was assessed in horizontal filter paper assays and recorded at different time points (N=30).

| **Balsam fir EO** | **Repellency (± SEM) %** | | | | |
| --- | --- | --- | --- | --- | --- |
| **% v/v** | **3** | **5** | **10** | **z** | **p** |
| 0.0 | 33.0±9.2 | 7.14±4.9 | 0.0±0.0 | - | - |
| 1.0 | 28.6±8.7 | 20.0±7.4 | 0.0±0.0 | 0.554 | 0.579 |
| 2.0 | 21.4±7.9 | 16.7±6.9 | 6.7±4.6 | 0.348 | 0.727 |
| 4.0 | 50.0±9.3 | 33.3±8.7 | 16.7±6.9 | 3.087 | **0.002** |
| 8.0 | 86.7±6.3 | 76.7±7.8 | 53.3±9.3 | 7.159 | **<0.001** |

**Supplementary Figure Legends**

**Figure S1.** The temperature regime in the three incubators mimicking different winter conditions. A) Cold winter temperatures. The incubator temperature was changed weekly as winter progressed. The bars represent the temperature set for that week. B) Warm winter temperatures. The temperature for the week is denoted by the bars. Warm and cold weeks alternated, but temperatures were always above 0^o^C. C) Fluctuating winter temperatures. The bars represent the daily (grey bars) and nightly (black bars) temperatures that were maintained for 1 week. Temperatures were typically warm (i.e. above 0^o^C), but ticks also experienced a series of cold snaps, with temperatures below 0^o^C.

**Figure S2.**  The time required to complete the righting reflex in ticks exposed to balsam fir needles for 2 weeks. Ticks exposed to balsam fir needles required more time to right themselves than did control ticks at 3 different winter temperature regimes 0^o^ C, 4^o^ C, and Fluctuating, -2^o^C to +2^o^C ). Sample sizes: 0^o^C control, N=19, balsam fir N=24; 4^o^C control N=21, balsam fir N=24; fluctuating temperature control N=21, balsam fir N=24. Bars represent the mean and error bars represent the standard deviation. The lines with an asterisk denote a significant difference. BF= Balsam Fir

**Figure S3.** Balsam Fir essential oil Chromatogram. Identity of corresponding peaks is listed in Table 1.

**Figure S4**. A higher concentration of β-pinene was required to kill ticks in open tubes compared with closed tubes. Chi-square (1)=35.0, p<0.00001). N=(closed 16 ticks/concentration, open 8 ticks/concentration).

**Supplementary Figures**

**Figure S1**

Figure S2


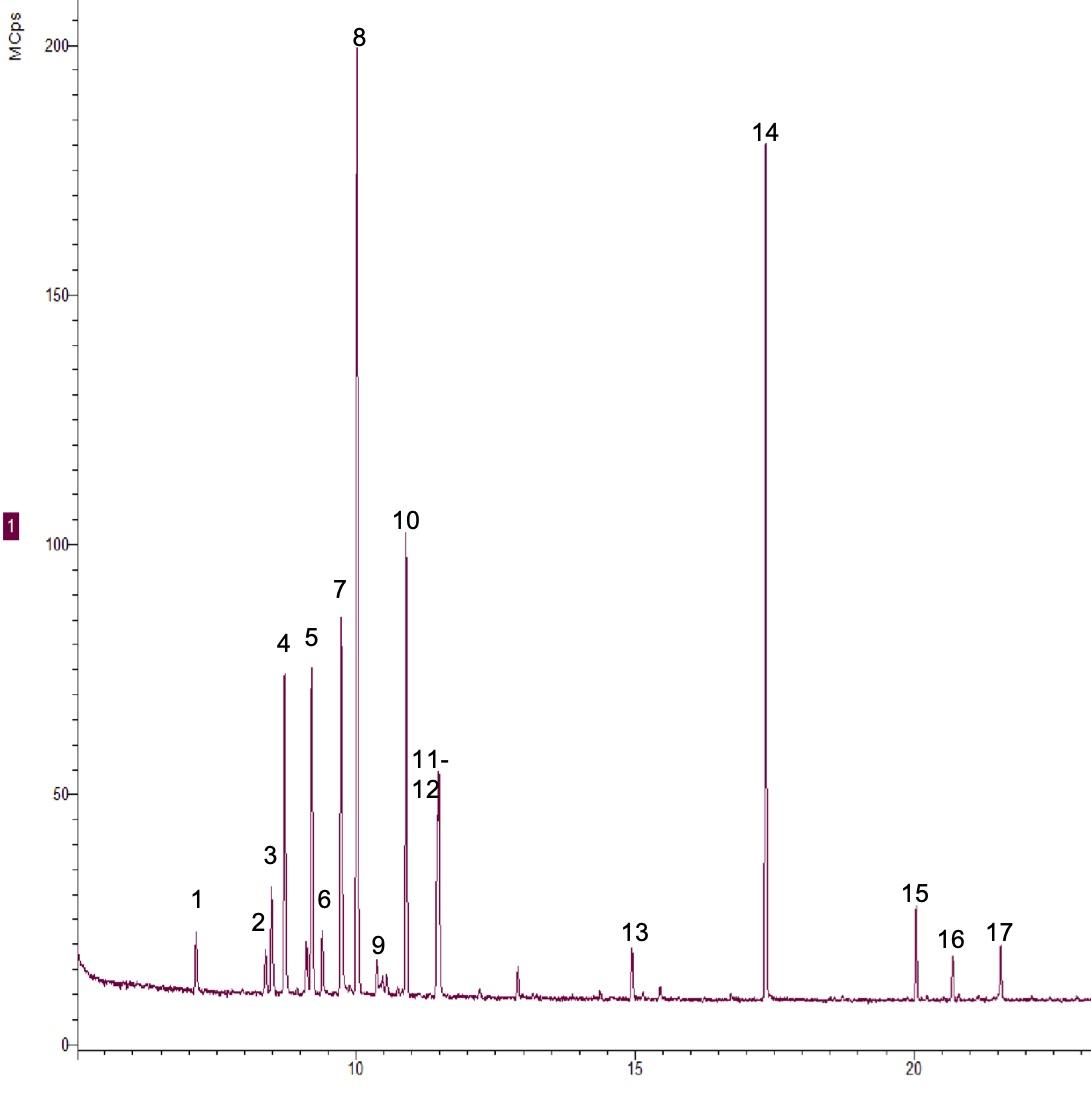


Figure S3.

**Figure S4**
